# Changes in Retinal Nerve Fiber Layer Thickness after Multiple Injections of Novel VEGF Decoy Receptor Conbercept for Various Retinal Diseases

**DOI:** 10.1038/srep38326

**Published:** 2016-12-06

**Authors:** Zhihua Zhang, Xiaolu Yang, Huiyi Jin, Yuan Qu, Yuan Zhang, Kun Liu, Xun Xu

**Affiliations:** 1 Department of Ophthalmology, Shanghai General Hospital, Shanghai Jiao Tong University School of Medicine, Shanghai, China

## Abstract

Conbercept is a recombinant fusion protein with high affinity for all vascular endothelial growth factor isoforms and placental growth factor. The repeated intravitreal injection of conbercept may cause intraocular pressure (IOP) fluctuations and long-term suppression of neurotrophic cytokines, which could lead to retinal nerve fiber layer (RNFL) damage. This retrospective fellow-eye controlled study included 98 eyes of 49 patients. The changes in IOP and RNFL thickness as well as the correlation between RNFL changes and associated factors were evaluated. The IOP value between the baseline and the last follow-up visit in the injection group and the IOP value of the last follow-up visit between the injection and non-injection groups were not significantly different (p = 0.452 and 0.476, respectively). The global average thickness of the RNFL (μm) in the injection group decreased from 108.9 to 106.1; however, the change was not statistically significant (p = 0.118). No significant difference in the average RNFL thickness was observed at the last follow-up visit between the injection and non-injection groups (p = 0.821). The type of disease was the only factor associated with RNFL thickness changes. In conclusion, repeated intravitreal injections with 0.05 mL conbercept revealed an excellent safety profile for RNFL thickness, although short-term IOP changes were observed.

Anti-vascular endothelial growth factor (VEGF) agents have been widely used for ocular vascular disorders. Recently, intravitreal injection of anti-VEGF agents has become the standard therapy for the treatment of patients with wet age-related macular degeneration (w-AMD)^1^ and is commonly used for the treatment of diabetic macular edema (DME)^2^. Therefore, the long-term safety of repeated anti-VEGF injections on the retinal nerve fiber layer (RNFL) has drawn attention. According to the latest meta-analysis, no association was observed between anti-VEGF injections and RNFL thickness changes^3^. However, those pooled studies mainly focused on ranibizumab (Lucentis; Genentech, Inc., South San Francisco, CA, USA) and bevacizumab (Avastin; Genentech, Inc.)^4^,^5^,^6^, which are monoclonal antibodies against VEGF-A^7^. Information about RNFL changes after other anti-VEGF injections is limited.

Conbercept (KH902; Chengdu Kanghong Biotech Co., Ltd., Sichuan, China) is a recombinant fusion protein designed as a receptor decoy with high affinity for all VEGF isoforms and placental growth factor (PlGF)^8^. Its efficacy following intravitreal injection has been proven *in vivo*^9^,^10^,^11^. A phase II, randomized, double-masked clinical trial has compared two dosing regimens including monthly injection (Q1M) and 3 consecutive monthly injection plus as-needed PRN treatment (3 + PRN), and it suggested that either treatment regimen was similarly efficacious^9^. Most ophthalmologists in China use the 3 + PRN regimen to treat patients with AMD^11^. The half-life of conbercept has not been calculated in human eyes, but in rabbit eyes is demonstrated to be 4.2 days, which is close to that of bevacizumab (4.3–6.61 days) and longer than that previously reported for ranibizumab (2.88–2.89 days)^12^. Because conbercept antagonizes two types of neurotrophic cytokines and because it has a higher binding affinity to VEGF and a longer half-life in the vitreous humor^12^, this agent might cause more RNFL damage than a VEGF-A inhibitor alone. Moreover, intraocular pressure (IOP) elevations immediately after intravitreal injection are known to occur^13^. Repeated injections may cause IOP fluctuations^13^ and lead to RNFL damage. In this study, we evaluated RNFL and IOP changes in patients receiving repeated conbercept injections and investigated the correlation between RNFL thickness changes and the associated factors.

## Results

### Characteristics of the Patients

Ninety-eight eyes of 49 patients (38 patients with w-AMD and 11 patients with DME) were enrolled in this study. Thirty-two (65.3%) patients were male, and 17 (34.7%) patients were female, with a mean age of 66 ± 9 (38–83) years. Only one eye of each patient received intravitreal conbercept injections, and the fellow eyes were included as the control group (non-injection group). The clinical characteristics of all the patients are listed in Table 1. All patients completed the 12 months follow-up period. No significant differences were observed in the baseline RNFL thickness and the IOP between the injection and non-injection groups. No serious complications such as endophthalmitis, sustained IOP elevation or marked anterior chamber reactions were noted during the follow-up period.

### IOP Changes

**Figure 1:**
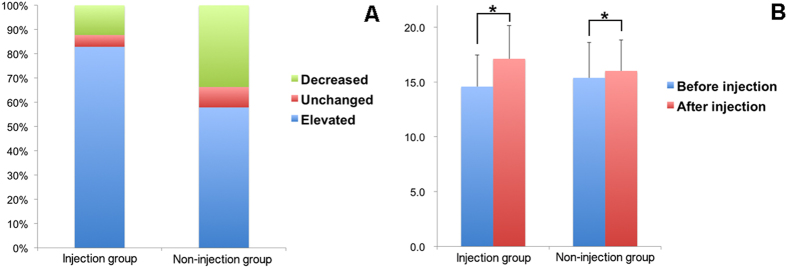
IOP change 30 min after each injection in the injection and non-injection groups. (**A**) In the injection group, the percentages of subjects who experienced an elevation in IOP, no change in IOP, or a decrease in IOP were 83%, 5 and 12%, respectively. The results were 58%, 8 and 34%, respectively, in the non-injection group. (**B**) The average IOP elevation was 2.54 mmHg in the injection group and 0.64 mmHg in the non-injection group. Both elevations were statistically significant; bars represent standard deviation (*p < 0.01).

A total of 392 injections were performed in 49 eyes. The IOP change after every injection in both groups was illustrated in Fig. 1. In the injection eyes, IOP elevation was noted after 83% of the injections, and the average IOP elevation after each injection was 2.54 mmHg. In the fellow eyes, IOP elevation was recorded after 58% of the injections, and the average IOP elevation after each injection was 0.64 mmHg. The IOP was significantly elevated after treatment in the injection group (p < 0.01). Additionally, the percentage of IOP elevation in the injection group was significantly higher than that in the non-injection group (p < 0.001).

**Figure 2:**
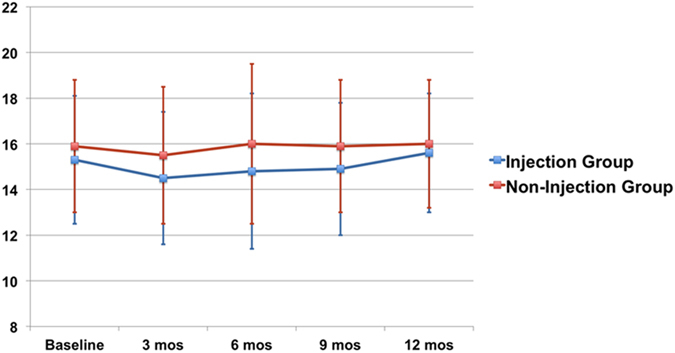
Changes in IOP from baseline through Month 12 in the injection and non-injection group. IOP was maintained stable overtime in both groups. There was no significant difference between the two groups for the mean IOP values throughout the follow-up; bars represent standard deviation.

In the injection group, the average IOP value of each month’s measurement and the IOP value of the last follow-up visit were 15.5 ± 2.9 and 15.6 ± 2.6 mmHg, respectively. In the non-injection group, the average IOP value of each month’s measurement and the IOP value of the last follow-up visit were 15.5 ± 3.0 and 16.0 ± 2.8 mmHg, respectively (Table 2). The IOP change between the baseline visit and the last follow-up visit in the injection group and the IOP value of the last follow-up visit between the injection and non-injection groups were not significantly different (p = 0.452 and 0.476, respectively). IOP changes from baseline through Month 12 in the injection group and non-injection group were illustrated in Fig. 2. IOP was stable overtime in both groups, and there was no significant difference between the two groups for the mean IOP values throughout the follow-up.

A spike in the IOP (IOP elevation ≥6 mmHg) was observed 31 times (7.9%) 30 min after injection in the injection group, and no spikes were observed in the non-injection group. The average IOP elevation of all spikes was 7.1 ± 2.1 mmHg (Table 2). Among these spikes, only one caused an IOP higher than 25 mmHg at 30 min after the injection, and the IOP returned to normal without any treatment at 60 min after injection. No sustained IOP elevation was detected during the follow-up period.

### Changes in RNFL Thickness

**Figure 3:**
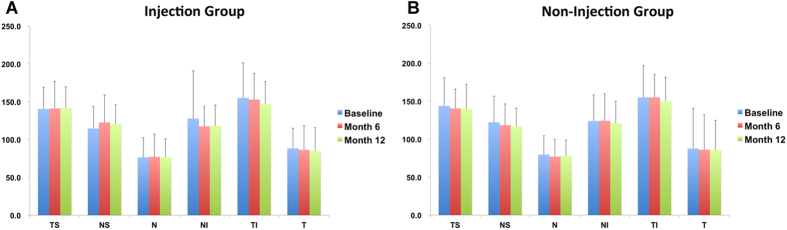
Sector assessment of RNFL thickness changes in the injection and non-injection groups. (**A**) In the injection group, no significant change was observed in RNFL thickness in any sector comparing baseline to Month 6 and Month 12. (**B**) In the non-injection group, no significant change in RNFL thickness in any sector was observed comparing baseline to Month 6 and Month 12; bars represent standard deviation.

The global average thickness of the RNFL in the injection group decreased from 108.9 ± 24.0 μm at baseline to 108.1 ± 26.1 μm at Month 6 (p = 0.741), and to 106.1 ± 19.4 μm at the last follow-up visit (p = 0.118). The global average thickness of the RNFL in the non-injection group decreased from 109.9 ± 31.4 μm at baseline to 108.3 ± 24.2 μm at Month 6 (p = 0.430), and to 107.0 ± 17.9 μm at the last follow-up visit (p = 0.255) (Table 2). However, the changes were not statistically significant. No significant difference in the average RNFL thickness was observed at the last follow-up visit between the injection and non-injection groups (p = 0.821). Regarding the sector assessment (temporal superior (TS), nasal superior (NS), nasal (N), nasal inferior (NI), temporal inferior (TI), and temporal (T)), no significant change in RNFL thickness in any sector in either group was observed comparing baseline to Month 6 and Month 12 (Fig. 3).

### Correlation Between RNFL Thickness Changes and Associated Factors

A significant decrease in RNFL thickness (>5 μm)^14^ was observed in 22.4% (11/49) of patients, accounting for 13.2% (5/38) of the AMD patients and 54.5% (6/11) of the DME patients. The number of IOP spikes, number of injections, type of disease, including AMD and DME, and patient age were included in the multiple logistic regression analysis. Considering the type of disease, patients with DME were more likely to have a decrease in RNFL thickness (p = 0.027) than patients with AMD. The other factors, including injection times, IOP spikes and patient age, were not associated with the change in RNFL thickness (Table 3).

## Discussion

Conbercept is a VEGF and PIGF dual antagonist and has become an increasingly common intervention for the treatment of retinal diseases in China. Thus, the safety of this novel drug is of great importance. To the best of our knowledge, this is the first study to investigate the RNFL thickness and IOP changes after repeated conbercept injections.

VEGF and PIGF were once regarded as specific angiogenic factors and currently are implicated in neuroprotection^15^,^16^. Theoretically, repeated conbercept injections may lead to IOP fluctuations and chronic suppression of neurotrophic cytokines, which can result in RNFL damage^17^,^18^. However, no significant change in RNFL thickness was observed comparing the initial visit to the Month 6 visit and the last follow-up visit in our study. Our result was consistent with previous studies focusing on anti-VEGF-A agents^5^,^6^. The decrease in RNFL thickness with increasing age is approximately 0.26 μm per year; therefore, the effect of a natural decrease in RNFL thickness during the follow-up period was negligible^19^. The reductions in RNFL thickness in AMD and DME patients in our study were 1.3 and 7.3 μm, respectively. The reduction in RNFL thickness in DME patients was significantly higher than that in AMD patients. Moreover, the logistic regression showed that patients with DME were more likely to have RNFL damage. Earlier studies have reported that inner retinal ischemia can cause ganglion cell death and axonal degeneration, resulting in an RNFL defect^20^,^21^,^22^. The RNFL loss in DME patients may be more closely related to vascular abnormalities and retinal ischemia^5^ than the anti-VEGF and anti-PIGF effects.

The mean IOP values were stable throughout the follow-up in both groups. However, at 30 min after injections, the mean IOP remained significantly above pre-injection values (p < 0.01). IOP spikes were observed 31 times (7.9%) in the injection group, but an IOP value higher than 25 mmHg after 30 min was only recorded once (0.3%). No patients required anti-glaucoma medication. Our results were consistent with previous reports on bevacizumab and Pegaptanib^23^,^24^. The general consensus is that a spike in IOP after intravitreal injection of anti-VEGF agents is common and is caused by the increased volume. In most cases, the spike is transient, and routine prophylactic use of IOP-lowering medications is not necessary^5^,^25^. Moreover, our study showed that repeated IOP fluctuations would not cause RNFL damage; however, our study did not include any patients with glaucoma. Future studies should examine whether the IOP spikes will harm the already compromised optic nerve in glaucoma patients. Moreover, we could only investigate the correlation between the change in RNFL thickness and the IOP change in this study, and future studies should analyze the correlation between the change in RNFL thickness and the change in the level of intravitreal neurotrophic cytokines.

The current study has several limitations. First, this study is a retrospective study, and the sample size is relatively small. Readers should consider that prospective studies with large sample sizes are needed to confirm our results. Second, we could not standardize the injection intervals because each patient had a different disease course. However, every patient completed the study through the 12-month follow-up period, and we were able to use the fellow eyes as the control group so that all the baseline characteristics were comparable. Additionally, we provided some direct results about the change in RNFL thickness after repeated intravitreal conbercept injections, which indicated that no significant RNFL loss occurred.

In conclusion, conbercept has an antagonistic effect for both VEGF and PIGF. Still, our study showed that repeated 0.05 mL intravitreal conbercept injections were associated with an excellent safety profile when considering the thickness of the RNFL, even though they could lead to short-term IOP changes.

## Methods

### Subjects

The study retrospectively analyzed the IOP and RNFL data of 49 patients who underwent more than three intravitreal conbercept injections for AMD or DME in one eye and were followed-up for 12 months between January 2010 and December 2014 at Shanghai General Hospital. The treatment indication for patients with AMD was untreated active subfoveal or juxtafoveal CNV. The treatment indication for patients with DME was untreated clinically significant DME. This study was performed in accordance with the tenets of the Declaration of Helsinki and was approved by the Ethics Committee of Shanghai General Hospital. Informed consent was obtained from all subjects. All patients had AMD or diabetic retinopathy in both eyes, but the fellow eyes did not require treatment and were observed as the control group (non-injection group). Intravitreal conbercept (0.5 mg/0.05 mL) injection was performed by one retinal specialist (KL). The exclusion criteria were as follows: (1) eyes with a history of glaucoma or suspected of glaucoma; (2) eyes with previous intravitreal injection of triamcinolone acetate, bevacizumab, ranibizumab or conbercept; (3) eyes with a history of treatment that could potentially affect the RNFL, such as cataract extraction, vitrectomy, and laser photocoagulation; (4) eyes combined with other ocular problems including neuro-ophthalmologic diseases, uveitis and refractive error exceeding  ± 6.00 diopters; and (5) the condition of the fellow eyes worsened and required laser photocoagulation and/or anti-VEGF injection. Each patient underwent a thorough ophthalmic examination, including best-corrected visual acuity (BCVA), slit-lamp observation, IOP and spectral domain optical coherence tomography (SD-OCT) measurements and fundus photography before the first injection and at each follow-up visit.

### IOP Measurement

The IOP values of the study and fellow eyes were analyzed 30 min before and after each injection and at each monthly visit for 12 months. An IOP spike was defined as an IOP elevation ≥6 mmHg after the injection compared with the IOP before the injection^5^. If an IOP elevation ≥10 mmHg after injection was noted, the patient was asked to stay another 30 min, and the IOP was measured at 60 min. If the IOP was higher than 30 mmHg at 60 min, a topical IOP-lowering medication treatment was prescribed, and the patient was reassessed after 24 hours.

### RNFL Thickness Measurement

An SD-OCT (Heidelberg Engineering, Heidelberg, Germany) examination was performed at baseline and at Month 6 and 12 to evaluate RNFL thickness by a single technician who was blinded to the patient information. The following parameters were used for each RNFL thickness acquisition: resolution mode: high speed; circle diameter: 3.5 mm; size X: 768 pixels (10.8 mm); size Z: 496 pixels (1.9 mm); scaling X: 14.05 μm/pixel; and scaling Z: 3.87 μm/pixel. The RNFL thickness from the inner margin of the internal limiting membrane to the outer margin of the RNFL layer was automatically segmented using SD-OCT software (Spectralis 5.1.3.0; Heidelberg Engineering, Heidelberg, Germany). The peripapillary RNFL thickness measurements of the global (G) average and six segments (TS, NS, N, NI, TI, and T) were analyzed automatically by the device.

### Other Outcome Measures

The correlations between the RNFL thickness change and potentially associated factors were analyzed. Considering the sample size, four factors that could most likely affect the RNFL after conbercept injection^5^ were included as follows: (1) IOP spikes; (2) number of injections; (3) type of disease, including AMD and DME; and (4) patient age. To determine the correlation, patients were divided into a group with a significant decrease in RNFL thickness and a group without a significant decrease in RNFL thickness. Regarding the test-retest variability, a change ≤5 μm in average RNFL thickness from baseline was defined as an acceptable change^14^.

### Statistical Analyses

Data analyses were completed using SPSS (Version 13.0, Chicago, IL, USA). Continuous variables (e.g., RNFL thickness and patient age) were expressed as the means and standard deviations. Categorical variables (e.g., sex) were expressed as numbers and frequencies. A paired t-test and Student’s t-test were used to compare RNFL thickness and the change in IOP within groups and between groups. Pearson chi-square and Mann-Whitney U tests were used for categorical data. For the associated factor analysis, logistic regression was performed to determine the odds ratio (OR) and 95% confidence interval (CI). A two-tailed p-value <0.05 was considered statistically significant.

## Additional Information

**How to cite this article**: Zhang, Z. *et al*. Changes in Retinal Nerve Fiber Layer Thickness after Multiple Injections of Novel VEGF Decoy Receptor Conbercept for Various Retinal Diseases. *Sci. Rep.* **6**, 38326; doi: 10.1038/srep38326 (2016).

**Publisher's note:** Springer Nature remains neutral with regard to jurisdictional claims in published maps and institutional affiliations.

## Figures and Tables

**Table 1:** Baseline characteristics of included patients who received more than three intravitreal conbercept injections for AMD and DME.

	Total	AMD	DME
Patient (eyes)	49 (98)	38 (72)	11 (22)
Injection/Non-injection (eyes)	49/49	38/38	11/11
Mean age	66 ± 9	69 ± 7	57 ± 9
Sex (male:female)	32:17	25:13	7:4
	Injection/Non-injection (p)	Injection/Non-injection (p)	Injection/Non-injection (p)
Baseline IOP (mmHg)	15.3 ± 2.8/15.9 ± 2.9 (0.301)	15.2 ± 2.9/15.8 ± 3.0 (0.357)	15.6 ± 2.5/16.1 ± 2.9 (0.658)
Baseline RNFL thickness (μm)	108.9 ± 24.0/109.9 ± 31.4 (0.863)	101.9 ± 13.8/101.3 ± 11.7 (0.823)	133.0 ± 35.0/139.6 ± 54.6 (0.738)

AMD: age-related macular degeneration.

DME: diabetic macular edema.

IOP: intraocular pressure.

RNFL: retinal nerve fiber layer.

**Table 2:** Outcomes of included patients who received more than three intravitreal conbercept injections for AMD and DME.

	Total	AMD	DME
Total number of injections	392	314	78
	Injection/Non-injection (p)	Injection/Non-injection (p)	Injection/Non-injection (p)
Average IOP (mmHg)	15.5 ± 2.9/15.5 ± 3.0 (0.887)	15.5 ± 2.9/15.4 ± 3.0 (0.641)	15.7 ± 2.8/15.9 ± 2.8 (0.473)
Final follow-up IOP	15.6 ± 2.6/16.0 ± 2.8 (0.476)	15.7 ± 2.7/16.2 ± 2.9 (0.450)	15.4 ± 2.5/15.5 ± 2.6 (0.947)
IOP spike number	31/0	27/0	4/0
IOP elevation during spike (mmHg)	7.1 ± 2.1 (6.0–16.6)	7.0 ± 2.1 (6.0–16.6)	7.7 ± 1.3 (6.3–9.4)
RNFL thickness (μm)			
Month 6	108.1 ± 26.1/108.3 ± 24.2 (0.971)	100.9 ± 12.1/102.1 ± 11.5 (0.656)	135.4 ± 43.8/131.7 ± 41.9 (0.849)
Final follow-up	106.1 ± 19.4/107.0 ± 17.9 (0.821)	100.6 ± 12.1/102.2 ± 11.9 (0.556)	125.3 ± 27.4/123.5 ± 25.0 (0.872)

AMD: age-related macular degeneration.

DME: diabetic macular edema.

IOP: intraocular pressure.

RNFL: retinal nerve fiber layer.

**Table 3:** Logistic regression analysis of factors associated with RNFL thickness.

Risk factors	IOP spike*	Injection number	Disease†	Patient age
OR	1.080	0.998	10.291	1.020
95% CI	0.446–2.615	0.739–1.348	1.309–80.884	0.924–1.125
p value	0.865	0.989	0.027	0.695

OR: odds ratio.

CI: confidential interval.

^*^IOP spike was defined as IOP elevation ≥6 mmHg after injection.

^†^Type of disease, including AMD and DME.
